# Supplementary material for: An Investigation of Left Ventricular Valve Disorders and the Mechano-Electric Feedback Using a Synergistic Lumped Parameter Cardiovascular Numerical Model
Source: Bioengineering (Basel). 2022 Sep 8;9(9):454. doi: 10.3390/bioengineering9090454 (PMC9495401; doi:10.3390/bioengineering9090454)
Supplement: Supplementary file 1 [file bioengineering-09-00454-s001.zip › bioengineering-1781153-supplementary.pdf]

Supplementary Materials: An Investigation of Left Ventricular Valve Disorders and the Mechano-Electric Feedback Using a Synergistic Lumped Parameter Cardiovascular Numerical Model

Nicholas Pearce * and Eun-jin Kim

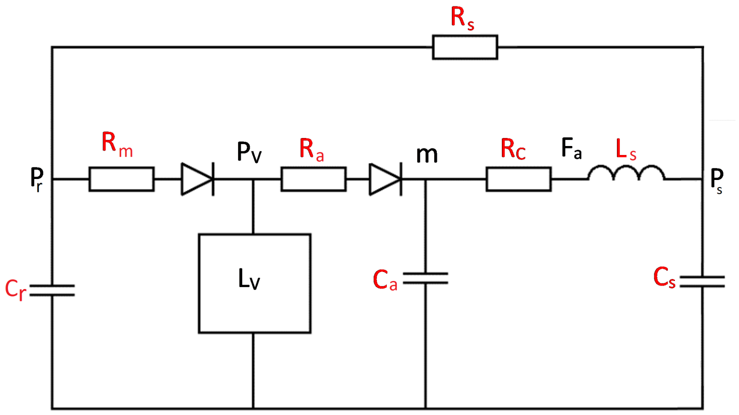

Figure S1. The equivalent electrical diagram of the complete cardiovascular system described. For abbreviation definitions refer to Table 3 in the main paper. The left atrium feeds blood at a pressure P_r through the mitral valve (the diode) into the left ventricle (L_v). As the blood passes through the valve it encounters a resistance R_m. With the mitral valve closed the aortic valve (second diode) opens allowing blood at pressure P_v to flow into aorta encountering a valve resistance R_a. The aorta has a compliance C_a. The aortic blood at pressure m flows (F_a) towards the remainder of the body encountering a characteristic resistance R_c. The blood has an inertia L_s due to its mass and encounters the resistance R_s and compliance C_s of the body's systemic arteries and tissues. The blood at pressure P_s after feeding the organs with fresh oxygen and nutrients is re-oxygenated and flows back to left atrium (with compliance C_r) ready for another cycle.

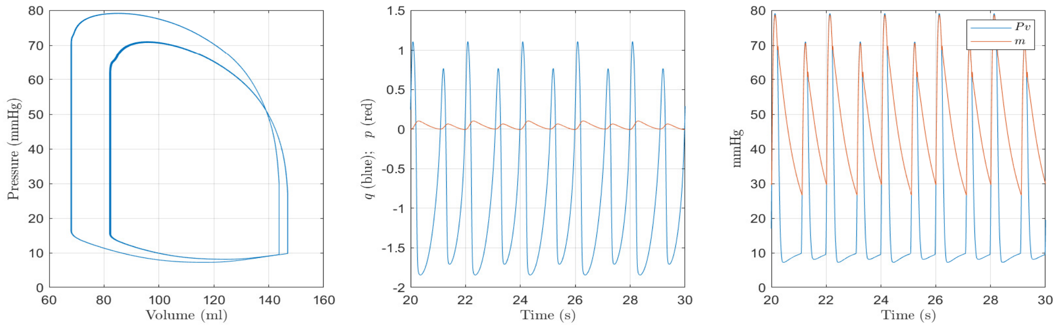

(a)$\mu_1 = 0.0128$

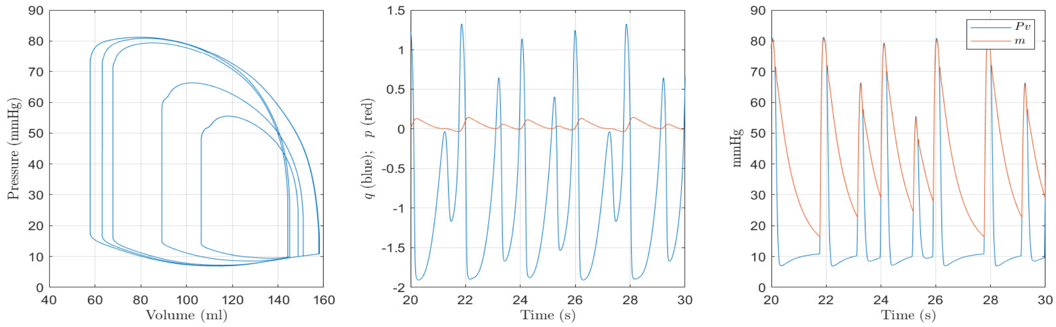

(b)$\mu_1 = 0.0145$

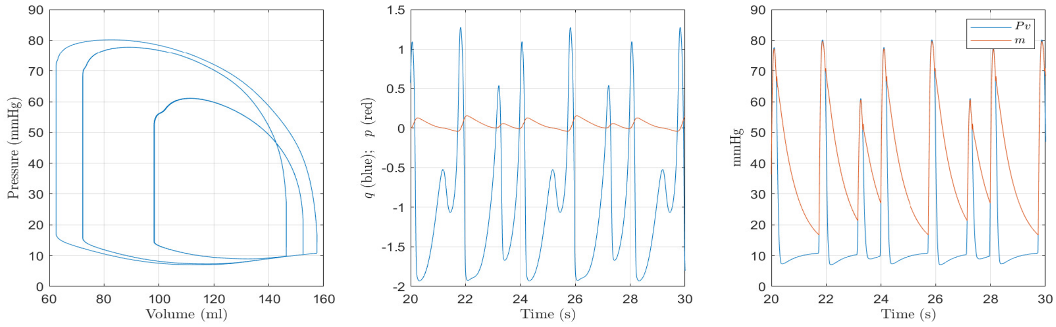

(c)$\mu_1 = 0.02$

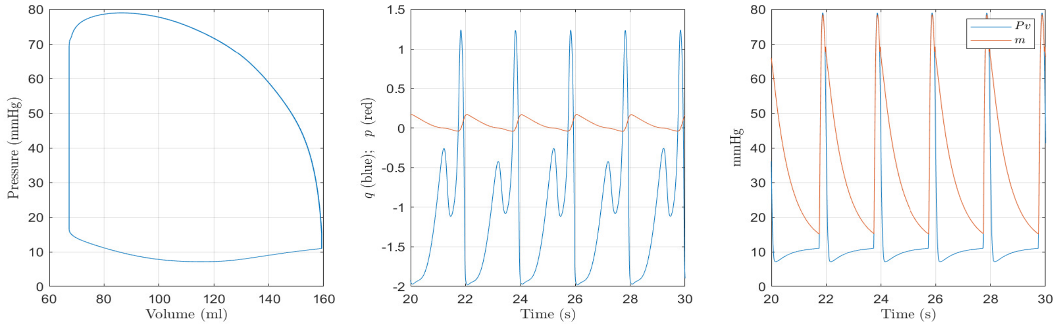

(d)$\mu_1 = 0.0268$

Figure S2. The effect of increasing μ_1 for the control case: $\mu_1 = [0.0128, 0.0145, 0.020, 0.0268]$.

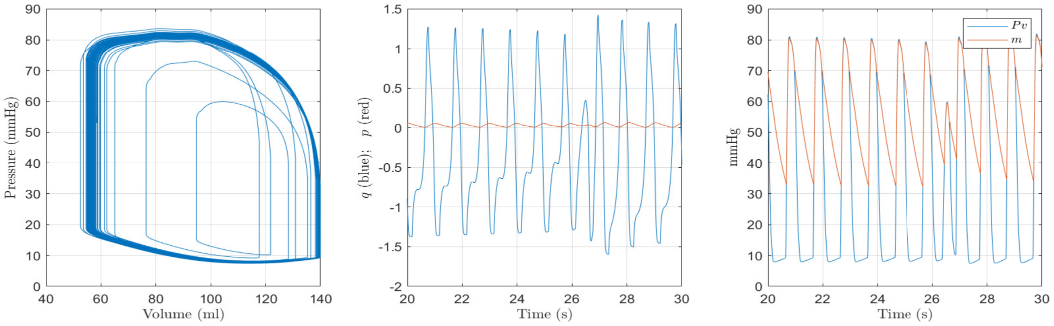

(a) $\mu_2 = 0.153$

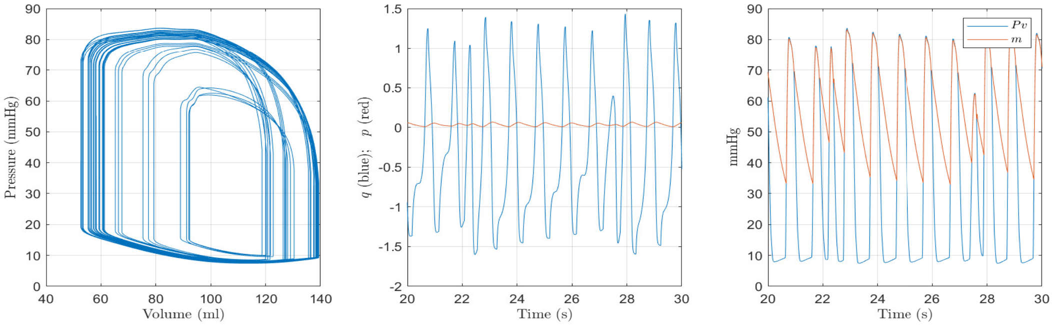

(b) $\mu_2 = 0.0.1584$

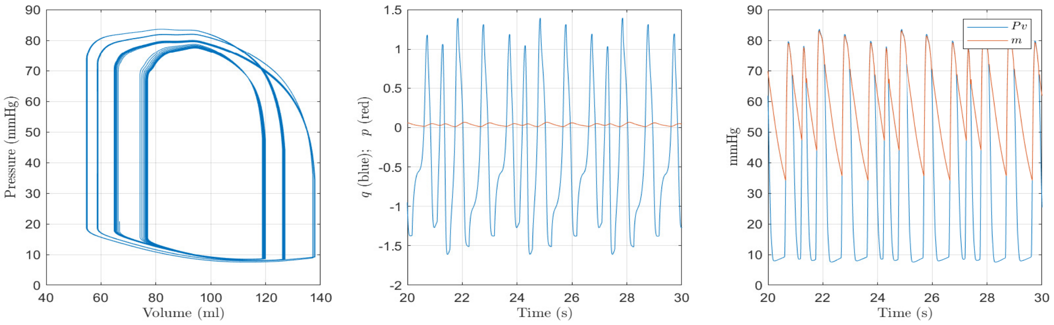

(c) $\mu_2 = 0.0.1718$

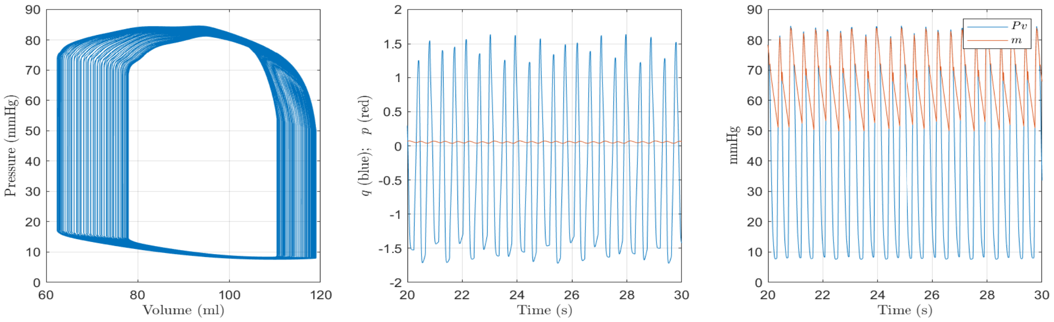

(d) $\mu_2 = 0.0.36$

Figure S3. *Cont.*

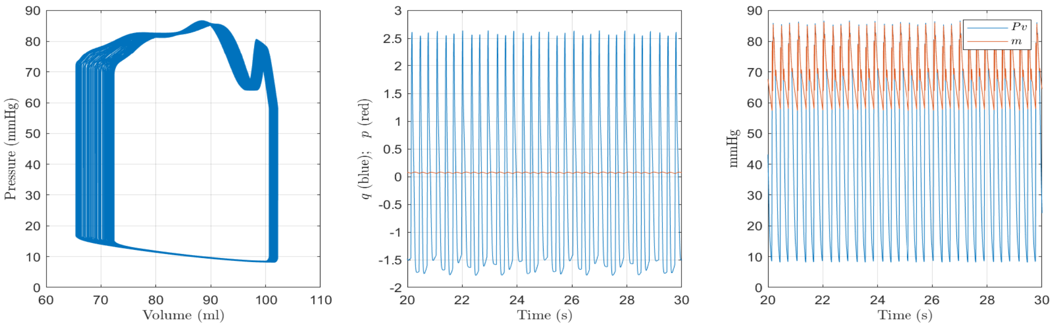

(a) $\mu_2 = 0.0235$

Figure S3. The effect of increasing μ_2 for the control case: $\mu_2 = [0.153, 0.1584, 0.1718, 0.36, 1.8]$.
